# Editorial: Non-pharmacologic Sustained Endothelial Shear Stress: An Evolving Clinical Paradigm

**DOI:** 10.3389/fphys.2021.790022

**Published:** 2021-11-16

**Authors:** Jose A. Adams, Alfredo Martínez

**Affiliations:** ^1^Division of Neonatology, Mount Sinai Medical Center, Miami Beach, FL, United States; ^2^Angiogenesis Group, Oncology Area, Center for Biomedical Research of La Rioja, Logroño, Spain

**Keywords:** pulsatile shear stress, whole body periodic acceleration (pGz), passive jogging device, exercise, enhanced external counter pulsation (EECP)

The endothelium plays an important role in the physiology and pathophysiology of a multitude of diseases. Whether the endothelium is the primary or secondary target in these diseases remains an open question. What is clear is that endothelial cells (EC) respond to mechanical signals to produce endogenous substances that signal to other cells in order to induce secondary signaling *via* endogenous proteins. The extension of the endothelium in the human body is so vast, that EC are a formidable target for therapeutic interventions which harness some of their beneficial output.

In this Research Topic, we broadly wanted to call the attention of the readership to how non-pharmacological interventions can modify EC biology. The signaling pathways which lead to the beneficial effects were explored using various clinically relevant models such as diabetes, muscular dystrophy, atherosclerosis, and congenital aortic valvar disease.

Adams et al. in their narrative review present an introduction to endothelial function in diabetes and the salient features of endothelial dysfunction in diabetes, followed by description of the concept of both pulsatile and laminar shear stress (SS) and endothelial output, most specifically endothelial derived nitric oxide (eNO). Interventions to induce pulsatile shear stress (PSS) on the human body are discussed, with physical exercise being the prototype of a non-invasive method to induce PSS. The non-invasive modalities to produce PSS were reviewed on the basis of the published literature. In addition to exercise, other non-invasive modalities to produce PSS are reviewed such as Enhanced External Counter Pulsation (EECP), Whole Body Vibration(WBV), Whole Body Periodic Acceleration (WBPA), and Passive Simulated Jogging (JD). All the aforementioned were reviewed with focus on their effects on diabetes. The authors provide a table of the currently known mechanical and biochemical effects of each modality, along with their effect on diabetes and ease of use (Table 1). They showed that the vasculoprotective, antioxidant, anti-inflammatory and glucose lowering effects of PSS can be harnessed, and form the basis of a paradigm shift for protection.

**Table 1:** The effects of non-pharmacologic interventions which increase pulsatile shear stress on selected diseases.

	**Interventions to increase Pulsatile Shear Stress**				**References**
	**Exercise**	**EECP**	**WBV**	**WBPA/JD**	
**Central nervous system**					
Stroke	+	+	unk	+	Martinez-Murillo et al., 2009; Belfiore et al., 2018; Park et al., 2018; Liu et al., 2019
Post cardiac arrest	±	unk	unk	+	Adams et al., 2003; Connolly et al., 2015
Parkinson's-disease	+	unk	±	+	Uhrbrand et al., 2015; Southard et al., 2018; Dincher et al., 2019
**Cardiovascular**					
Hypertension	+	+	+	+	Cornelissen and Smart, 2013; Wong et al., 2016; Sackner et al., 2019; Liang et al., 2020
Myocardial infarction	+	+	+	+	Miyamoto et al., 2011; Anderson et al., 2016; Qin et al., 2016; Shekarforoush and Naghii, 2019
Peripheral artery disease	+	+	+	+	Rokutanda et al., 2011; Martin et al., 2012; Lane et al., 2017; Mahbub et al., 2019
**Pulmonary**					
Asthma	+	unk	unk	+	Abraham et al., 2006; Eichenberger et al., 2013
COPD	+	+	+	+	Paneroni et al., 2017; Zhou et al., 2018; Sabater et al., 2019; Zhao et al., 2020
Pulmonary hypertension	+	unk	+	+	Adams et al., 2000; Gerhardt et al., 2017; Morris et al., 2017
**Infection-Sepsis**	+	unk	±	±	Alawna et al., 2020; Sackner and Adams, 2020; Sanudo et al., 2020

*The beneficial effects of various non-pharmacologic interventions which modify pulsatile shear stress; Exercise, Enhanced External Counterpulsation (EECP), Whole Body Vibration (WBV), and Whole Body Periodic Acceleration or Passive Simulated Jogging (WBPA/JD) on selected diseases. A beneficial effect (+), unknown (unk), possible or highly likely (±). Selected references were obtained first from the most recent meta-analysis or systematic review, then from clinical studies or compelling animal data, and lastly review data*.

Signaling pathways induced by PSS using WBPA was utilized by Uryash et al. in a genetic model of Duchenne muscular dystrophy (DMD, dystrophin deficiency) cardiomyopathy. In an elegant fashion, using *in vitro* ion selective microelectrodes, these investigators showed the intracellular effects of WBPA. Furthermore, they showed that exposing mice to WBPA for 1 hr per day, 5 days per week, for 3 months, decreased intracellular calcium dyshomeostasis, reactive oxygen species (ROS) production and improved cell viability and cardiomyocyte contractility. These findings were coupled with genomic upregulation of Utrophin, which improves muscular contractility in DMD (Perkins and Davies, 2002; Soblechero-Martin et al., 2021). The changes were abolished by nitric oxide synthase inhibition prior to WBPA, suggesting NO is operative in the signaling pathway of WBPA. There are clinical implications to this work, since exercise is not a viable strategy in these patients due to severity of muscle weakness. WBPA provides a potential non curative but simple therapeutic strategy.

Antequera-González et al. reviewed the link between endothelial dysfunction (ED) and congenital bicuspid aortic valve (BAV). BAV has been shown to be associated with congenital genetic abnormalities in various signaling pathways [Notch-1, Roundabout guidance receptor 4 (ROBO4), GATA binding protein 4(GATA4)]. Disturbed flow and wall shear stress patterns in the aorta of BAV subjects was shown to induce ED. The review addresses the question of the contribution of genetic abnormalities and hemodynamic alterations in ED observed in BAV. Figure 2 of the manuscript provides a detailed hypothesis driven model of how each can be contributory to ED. The authors enumerate potential therapeutic interventions such as; endothelial progenitor cells, endothelial colony forming cells, pharmacologic therapy [angiotensin-converting enzyme 1 (ACE1) inhibitors, statins, antioxidants], and gene silencing therapy (miRNA). The latter have not been validated in ED induced by BAV. It is plausible that some of the mechanical interventions proposed in the manuscript by Adams et al. could also improve endothelial function in BAV, since WBPA, EECP, exercise, and WBV have been used in patients with ED and found to improve flow mediated vasodilation (Sakaguchi et al., 2012; Gurovich and Braith, 2013; Takase et al., 2013; Beck et al., 2014; Ashor et al., 2015; Jaime et al., 2019; Xu et al., 2020).

Wang et al. reviewed the link among primary cilia on endothelial cells, blood flow, and atherosclerosis. It has been established that endothelial cells lining blood vessels where the blood follows a laminar flow usually lack primary cilia. On the other hand, endothelial cells located in areas with turbulent or oscillatory flow present a higher number of these sensory cellular elements. The presence of primary cilia protects the endothelium and cardiovascular health by mediating several intracellular and extracellular signaling pathways, including transforming growth factor (TGF), intracellular Ca^2+^ and nitric oxide. Undoubtedly, shear stress is a central mechanism in the physiology of the cardiovascular system, and external artificial modifications of this physical parameter may constitute a novel approach to improve the cardiac health of the population and mitigate cardiovascular accidents.

It is clear, from the aforementioned works in this Research Topic, that the endothelium provides a therapeutic target for a myriad of diseases. This Research Topic provided a tantalizing insight into EC in diabetes, congenital muscle disease, and genetic valvulopathy (BAV) and some therapeutic interventions using PSS. However, PSS as a therapeutic intervention may also find its way to other diseases, which are enumerated on Table 1.

We are grateful to the contributions of the various authors in putting forward their insightful work, and the hard work of the reviewers. Most grateful to our friend and mentor, the late Professor Marvin Sackner, **RIP**, who coined the phrase “Movement is Everything” as it pertains to the benefits of exercise/PSS in daily living and disease management.

## Author Contributions

All authors listed have made a substantial, direct and intellectual contribution to the work, and approved it for publication.

## Conflict of Interest

JA draws no salary from Sackner Wellness Products LLC a company that has a patent on a passive jogging device. He owns 20% of the domestic and foreign patents. The remaining author declares that the research was conducted in the absence of any commercial or financial relationships that could be construed as a potential conflict of interest.

## Publisher's Note

All claims expressed in this article are solely those of the authors and do not necessarily represent those of their affiliated organizations, or those of the publisher, the editors and the reviewers. Any product that may be evaluated in this article, or claim that may be made by its manufacturer, is not guaranteed or endorsed by the publisher.
